# The Influenza Virus RNA-Polymerase and the Host RNA-Polymerase II: RPB4 Is Targeted by a PB2 Domain That Is Involved in Viral Transcription

**DOI:** 10.3390/v14030518

**Published:** 2022-03-03

**Authors:** Jessica Morel, Laura Sedano, Nathalie Lejal, Bruno Da Costa, Eric Batsché, Christian Muchardt, Bernard Delmas

**Affiliations:** 1 Unité de Virologie et Immunologie Moléculaires, INRAE, Université Paris-Saclay, 78350 Jouy-en-Josas, France; jessica.morel@inrae.fr (J.M.); laura.sedano@inrae.fr (L.S.); nathalie.lejal@inrae.fr (N.L.); bruno.dacosta@inrae.fr (B.D.C.); 2 Biological Adaptation and Ageing (B2A), Institut de Biologie Paris-Seine (IBPS), Sorbonne Université, CNRS, 75005 Paris, France; eric.batsche@sorbonne-universite.fr (E.B.); christian.muchardt@sorbonne-universite.fr (C.M.)

**Keywords:** influenza virus, RNA-polymerase, RNA-polymerase II, protein-protein interaction, PPI, cap snatching, transcription, binary complementation assay

## Abstract

Influenza virus transcription is catalyzed by the viral RNA-polymerase (FluPol) through a cap-snatching activity. The snatching of the cap of cellular mRNA by FluPol is preceded by its binding to the flexible C-terminal domain (CTD) of the RPB1 subunit of RNA-polymerase II (Pol II). To better understand how FluPol brings the 3′-end of the genomic RNAs in close proximity to the host-derived primer, we hypothesized that FluPol may recognize additional Pol II subunits/domains to ensure cap-snatching. Using binary complementation assays between the Pol II and influenza A FluPol subunits and their structural domains, we revealed an interaction between the N-third domain of PB2 and RPB4. This interaction was confirmed by a co-immunoprecipitation assay and was found to occur with the homologous domains of influenza B and C FluPols. The N-half domain of RPB4 was found to be critical in this interaction. Punctual mutants generated at conserved positions between influenza A, B, and C FluPols in the N-third domain of PB2 exhibited strong transcriptional activity defects. These results suggest that FluPol interacts with several domains of Pol II (the CTD to bind Pol II), initiating host transcription and a second transcription on RPB4 to locate FluPol at the proximity of the 5′-end of nascent host mRNA.

## 1. Introduction

Influenza A viruses (IAVs) are important viral respiratory pathogens of humans and are members of the Orthomyxoviridae family. These viruses contain a negative-sense single-stranded segmented RNA genome (reviewed in [1]). The three largest segments encode the subunits of the viral RNA-dependent RNA-polymerase (FluPol), including the two basic proteins PB1 and PB2 and the acidic subunit PA [2]. Unlike what has been observed for many other RNA viruses, the IAV genome is transcribed and replicated in the nucleus of infected cells, wherein the FluPol nucleotide polymerization activity carries out both replication and transcription. During the latter, an additional “cap-snatching” function is implemented to steal short 5′-capped RNA primers from host mRNAs [3].

In the viral particle, the eight viral RNA genomic segments are packaged together with numerous copies of the viral nucleoprotein (NP) and of FluPol in viral ribonucleoprotein complexes (vRNPs). After binding to sialic acid on the cell surface, virions are endocytosed and fused with the endosomal membrane. vRNPs are then released into the cytoplasm and transported to the nucleus where they start synthesizing viral mRNAs to produce viral proteins. Thus, FluPol transcribes each of the vRNA genome segments, producing positive sense viral mRNA with a 5′-terminal N7-methyl guanosine (m7G) cap and a 3′-polyA tail. FluPol which does not possess capping activity binds to m7G on nascent host RNAs using a cap-binding domain on PB2. Then, it cleaves the host mRNA approximately 10- to 15 nucleotides downstream of the 5′-cap, using the PA endonuclease. The resulting short 5′ capped RNA fragment acts as a primer to initiate transcription of the viral genome segments. Polyadenylation is carried out by a FluPol stuttering at a 5′-proximal poly(U) polyadenylation signal present on each genomic vRNA [4]. The complete FluPol transcription activity (initiation, elongation, polyadenylation, and FluPol recycling) has been characterized at near atomic resolution [5,6].

Cap snatching by FluPol requires interaction(s) with the host transcriptional machinery [7,8,9]. The host RNA-polymerase II (Pol II), a complex of 12 subunits (RPB1-12), synthesizes cellular mRNAs (and diverse noncoding RNAs) which are 5′-m7G capped. Its catalytic center is borne by the two largest subunits (RPB1 and RPB2), while smaller subunits (RPB3 to RPB12) are arrayed at the periphery [10,11]. RPB1 in mammals has a large mobile C-terminal domain (CTD) composed of 52 heptad repeats (consensus sequence Tyr-Ser-Pro-Thr-Ser-Pro-Ser) that recruit factors required for RNA splicing and termination. Residues of the CTD are subjected to post-translational modifications, the most studied being the phosphorylation of Ser2 and/or Ser5. The Ser5P CTD is recognized by the nuclear machinery involved in early steps of mRNA transcription, but remains detectable throughout the gene body [12]. FluPol interacts with RPB1 by binding to its CTD, and more specifically to heptad repeats carrying the Ser5P modification [13]. This interaction has been structurally defined with CTD mimic Ser5P peptides and FluPols of influenza A, B and C viruses [14,15]. These studies document a bipartite binding site on the CTD, while the involved FluPol surfaces appear imperfectly conserved between influenza virus subtypes.

In this study, to better define the molecular mechanisms underlying the FluPol cap-snatching activity, we explored whether FluPol may interact with Pol II via domains or subunits other than the CTD of RPB1. We thus used binary complementation assays to systematically investigate effective interactions between FluPol and Pol II subunits. This approach allowed for identification and validation of an interaction between the third N-ter domain of PB2 and RPB4. This interaction was conserved among FluPol virus types, and mutations in the implicated domain of the viral protein were found to cripple viral transcription. These results suggest that during viral transcription, FluPol associates with the regulatory CTD domain of RPB1, while also interacting with RPB4, at a site where nascent capped RNAs emerge from Pol II. We speculate that this second complementary anchoring position may participate in the docking of FluPol onto nascent RNAs as a step preliminary to the “cap-snatching”.

## 2. Materials and Methods

### 2.1. Cells and Virus

HEK-293T cells were maintained in Dulbecco’s modified Eagle’s Medium (DMEM) supplemented with 10% fetal calf serum (FCS), 2 mM L-glutamine, 100 IU/mL penicillin, 100 mg/mL streptomycin at 37 °C and 5% CO_2_. Wild-type (wt) and PB2 mutant viruses were generated by reverse genetics using the 12-plasmid reverse genetics system kindly provided by G. Brownlee [16]. Site-directed mutagenesis was carried out on the PB2 gene by using the QuikChange II site-directed mutagenesis kit (Agilent Technologies France, Les Ulis, France). The viruses were prepared as previously described [17]. Briefly, a 1-day coculture of HEK-293T and MDCK cells (seeding of 3 × 10^5^ and 4 × 10^5^ cells, respectively, in P6 plates) was transfected with a plasmid mixture (0.25 μg per plasmid) using Fugene HD (Promega France, Charbonnières-les-bains, France) according to the manufacturer’s recommendations. At 48 h post-transfection, cell supernatants were harvested and used to inoculate MDCK cells for the production of rescued virus stocks. The FluPol genes of the recombinant viruses were sequenced to validate the presence of engineered mutations.

### 2.2. Protein-Protein Interaction Assays

#### 2.2.1. Split-Luciferase Complementation Assay

**Figure 1:**
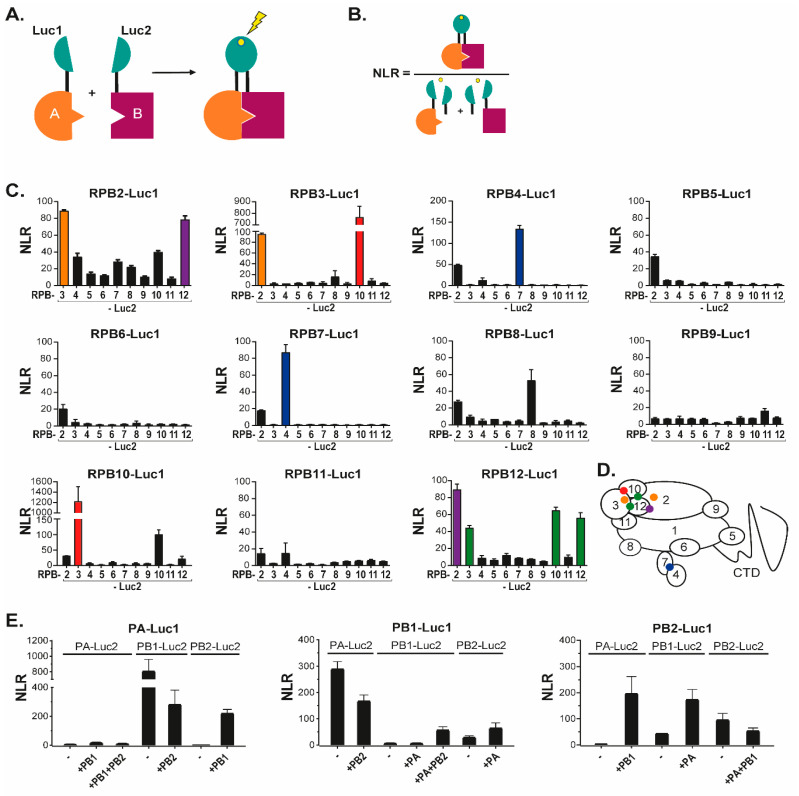
The split-luciferase interaction assay and its validation with Pol II and FluPol subunits. (**A**) Schematic of the principle of split-luciferase complementation assay. If protein A and protein B interact, the activity of luciferase is restored and luminescence will be emitted in presence of its substrate. (**B**) The normalized luminescence ratio (NLR) is calculated as described. (**C**) NLR values obtained for pairs of Luc1/Luc2-tagged Pol II subunits co-expressed into HEK-293T cells. Twenty-four hours after transfection of plasmids driving Pol II subunits expression, cells were lysed and luminescence was measured. Colors indicate reciprocated interactions, orange for RPB2-RPB3 interaction, red for RPB3-RPB10 interaction, blue for RPB4-RPB7 and violet for RPB2-RPB12. The green color indicates one-way interaction detection, RPB12 with RPB3 and RPB10. (**D**) Interactions are represented by a colored dot on a scheme of Pol II and its subunits as defined in (**C**). (**E**) NLR values obtained for pairs of Luc1/Luc2-tagged FluPol subunits plasmids transfected into HEK293T cells with/without untagged FluPol subunits. Data are mean ± s.d. n = two technical replicates representative of three independent experiments.

A first protein-protein interaction assay was based on the complementation of two trans-complementing fragments of *Gaussia princeps* luciferase (Gluc), Luc1 and Luc2, as described in Cassonnet et al. [18]. Interaction-mediated luciferase activity was measured in cultured cells transiently expressing a protein fused to Gluc1 and another one fused to Gluc2. Codon-optimized of human Pol II cDNAs encoding subunits RPB3, RPB4, RPB5, RPB6, RPB7, RPB8, RPB9, RPB10, RPB11 and RPB12 (all with a HA-tag at their N-terminus) were cloned in pCI-LL-Luc1 and pCI-LL-Luc2 vectors [19], thus resulting to the in frame fusion of the HA-tagged Pol II open reading frame subunits with the Luc1/Luc2 moieties. In these constructs, Pol II subunits are separated from the Luc1/Luc2 moieties by a 10-amino acid long linker (sequence GGGGSGGGGS). The plasmid encoding the RPB2 subunit under the control of the CMV promoter (kindly provided by Benoit Coulombe) was used as a template to insert at the RPB2 3′-end the LL-Luc1/Luc2 moieties. The plasmid pFLAG-Pol-II WT encoding human RPB1 (Addgene number 35175, deposited by Benjamin Blencowe) was used to generate two pCI-LL-Luc1/Luc2 derivates encoding 4 repeats (4 × YSPTSPS) of the CTD tail fused to Luc1 or Luc2. FluPol PA, PB1 and PB2 subunits cDNA cloned in pCI-LL-Luc1/Luc2 vectors were kindly provided by Nadia Naffakh [19]. Influenza B and C PB2 cDNAs (which were kindly provided by Wendy Barclay and Sylvie van der Werf, respectively), were used to subclone in frame their 5′-domains with Luc1 in pCI-LL-Luc2 with the Gibson Assembly Master Mix Kit (New England BioLabs, Ipswich, Massachusetts, USA). Twenty-six pCI-LL-Luc1/Luc2 derivates encoding cellular proteins irrelevant in FluPol-Pol II interaction were kindly provided by Caroline Demeret. Point mutations and deletions in FluPol cDNAs were generated using Q5 Site-Directed Mutagenesis Kit (New England BioLabs, Ipswich, MA, USA). HEK-293T cells were seeded at a concentration of 10^5^ cells per well in 48-well plates. Twenty-four hours post seeding, cells were transfected in duplicate/triplicate with the indicated combinations of pCI-derived plasmids by using polyethylenimine (Polyscience Inc., Le Perray-en-Yvelines, France). In each well, 150 ng of each pCI-LL-Luc1/Luc2 derivate are co-transfected with 150 ng of an empty pCI vector. In several assays, the empty pCI vector was replaced by pCI vectors encoding wild-type FluPol subunits. Twenty-four hours post-transfection, cells were lysed using the *Renilla* luciferase assay buffer (Promega France, Charbonnières-les-bains, France) for 30 min at room temperature. Next, the luciferase enzymatic activity recovered by the assembly of the Luc1 and Luc2 subunits was measured using *Renilla* luciferase assay reagent and a Tecan Infinite 200 PRO luminometer (*Renilla* luminescence counting program; integration time of 10 s after injection of 50 μL of the reagent). Normalized luminescence ratios (NLRs) were calculated using pGluc1 and pGluc2 plasmids only encoding *Gaussia* luciferase moieties as described previously by Cassonnet et al. [18]. The NLR for a given interacting protein pair A-B was calculated by dividing the luminescent signal by the sum of the luminescent measured in control cells as indicated in Figure 1A,B. Significance of NLR signals from the noise was determined using GraphPad to identify outliers with a threshold of 1%.

#### 2.2.2. Bimolecular Fluorescence Complementation (BiFC) Assay

BiFC constructs were based on the complementation of two trans-complementing fragments of Venus fluorescent protein, Venus1 (amino acids 1 to 155) and Venus2 (amino acids 156 to 239), as described by Shyu et al. [20]. Interaction-mediated fluorescence is observed in cultured cells when a protein A fused to Venus1 interacts with a protein B fused to Venus2. Thus, Pol II and FluPol subunits cDNAs were cloned using adequate restriction sites in pCI-LL-Venus1 and -Venus2 vectors, thus resulting to the in frame fusion of the cloned open reading frames followed by a 10-amino acid long linker (sequence GGGGSGGGGS) with the Venus1/2 moieties. Cells were seeded on coverslips in P12-wells and transfected 24 h later with combinations of pCI-LL-Venus1/2 derivates (1 µg/plasmid). Twenty-four hours after transfection, cells were fixed using 4% paraformaldehyde for 10 min and permeabilized for 15 min using 0.1% Triton X-100. Nuclei were stained with DAPI. The excitation peak used to reveal Venus fluorescence was 515 nm and its emission was revealed at 528 nm. Images were captured with a confocal microscope and processed with the ImageJ software.

### 2.3. Minireplicon and Transcription Assays

#### 2.3.1. Minireplicon Assay

HEK-293T cells were seeded in P96 wells (5 × 10^4^ cells/well) and transfected one day later with plasmids driving expression of wild-type or mutated forms of the PA, PB1, PB2, and NP viral proteins with the plasmid pPolI-WSN-NA-firefly luciferase as previously described [21]. Plasmid pMAX-GFP (Lonza, Walkersville, MD, USA) was used as an internal control for transfection efficiency and data normalization. As a negative control, 293T cells were transfected with polyethylenimine (PEI) with the same plasmids minus the PA expression plasmid. Forty-eight hours post-transfection, cells were lysed in 150 µL PLB buffer (30 mM Tris pH7.9, 10 mM MgCl_2_, 1% Triton X-100, 20% Glycerol, 1 mM DTT). Luminescence activity was measured with Luciferase Assay System (Promega) on a Tecan Infinite M200Pro luminometer according to the manufacturer’s instructions. Replicon activity was quantified by a ratio between the luminescence and the fluorescence signals.

#### 2.3.2. Transcription Assay

To quantify transcription activity of FluPol mutants, the same procedure than described in the minireplicon assay was used, except that the wild-type PA subunit was replaced by the PA C95A mutant that is deficient for replication [22].

### 2.4. Co-Immunoprecipitation Assay

HEK-293T cells plated in 6-well plates were transiently transfected with 1 μg of pci-PB2(36-247)-FLAGtag and/or with 1 μg pci-HAtag-RPB4 using PEI. Twenty-four hours post-transfection, cells were rinsed and scrapped at 4 °C into 1 mL of 50 mM Tris-HCl pH7.4, 2 mM EDTA, 150 mM NaCl, 1% Triton X-100, 10% glycerol supplemented with protease inhibitor (Thermo Fisher Scientific, Les Ulis, France). After 1 h, this material was centrifugated (20 min, 12,000× *g* at 4 °C). Supernatants were collected and incubated under gentle agitation overnight at 4 °C with 80 μL of a 1:1 slurry of sepharose-IgG beads (GE Healthcare, Chicago, IL, USA) previously coated with anti-FLAG M5 antibody (Sigma-Aldrich, St. Louis, MO, USA). The beads were washed three times with 1 mL of lysis Buffer and once with PBS, then treated for 2 min at 100 °C in Laemmli’s denaturating buffer plus 5% 2-mercaptoethanol, and centrifugated. Resulting supernatants were subjected to SDS-PAGE followed by a western blot (WB) analysis. An anti-HA antibody conjugated with peroxydase (Roche Diagnostics GmbH, Mannheim, Germany) and an anti-FLAG antibody conjugated with peroxydase (Sigma-Aldrich, Darmstadt, Germany) were used to reveal immunoprecipitated products in the WB.

## 3. Results

### 3.1. Novel Interactions between FluPol and Pol II Subunits

To identify novel sites of interactions between FluPol and Pol II subunits, we used the previously described *Gaussia princeps* luciferase-based complementation assay [18,23]. In this assay, an interaction between two proteins each fused to either the Luc1 or the Luc2 segments of the *Gaussia* luciferase enzyme, results in reconstitution of a functional luciferase activity, which can be quantified by addition of substrate (Figure 1A). To favor proper folding of the FluPol and Pol II subunits, the luciferase segments were fused onto their C terminus. For each sample, the specificity of the interaction over background was estimated by calculating a normalized luminescent ratio (NLR—Figure 1B).

To validate the assay, we first fused each Pol II subunit (from RPB2 to RPB12) with either Luc1 or Luc2, then co-expressed the Luc1-fusions with each Luc2-fusion (Figure 1C). This approach identified strong and reciprocal contacts between RPB2 and RPB3, RPB2 and RPB4, RPB2 and RPB12, RPB3 and RPB10, and RPB4 and RPB7. Interaction signal was also identified between RPB12 and RPB3 or RPB10, albeit in a non-reciprocal manner. Finally, RPB8, RPB10, and RPB12 were found to interact with themselves. Confrontation of these observations with the 3D-structure of Pol II [24] illustrated that the luciferase complementation assay was very efficient at detecting direct interactions between Pol II subunits, while it was oblivious to interactions mediated by the Pol II complex, possibly due to the shortness of the GGGSGGGS linker (summarized by the color code in Figure 1D).

We next used a similar approach to monitor interactions between FluPol subunits (Figure 1E). In these experiments, co-expressed Luc1/Luc2-tagged forms of PA and PB1 yielded NLR signal both in the presence, and in the absence of untagged PB2. In contrast, interaction of PB2 with PA or PB1 was detected only when the three FluPol subunits were co-expressed. As for Pol II, these interactions between FluPol subunits were in agreement with the known 3D-structures of the viral enzyme and also matched earlier observations on the sequential assembly of in the cytoplasm and in the nucleus [11,25,26].

**Figure 2:**
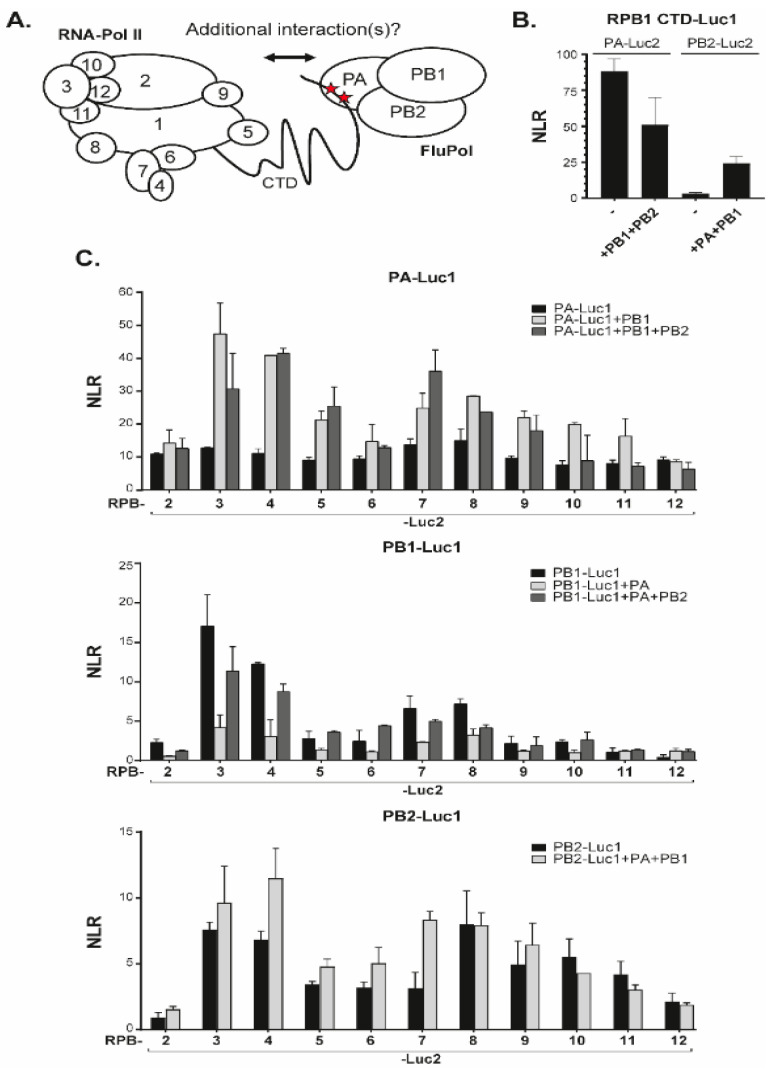
Split-luciferase assays between FluPol and Pol II subunits. (**A**) Schematic of FluPol and Pol II with their subunits and the interaction between the CTD of Pol II with FluPol. Red stars indicate the YSPTSpPS CTD motif (with the phosphorylated Serine 5). (**B**) A plasmid encoding a CTD stretch of RPB1 fused the Luc1 moiety was co-transfected with plasmids encoding PA or PB2 fused to the Luc2 moiety. Plasmids encoding untagged PA, PB1, or PB2 were also added when indicated to produce complete FluPol. Twenty-four hours post-transfection, cells were lysed and luminescence signal was measured. (**C**) Pairs of plasmids encoding a Luc1-FluPol subunit with a Luc2-Pol II subunit were transfected into HEK 293T along with/without untagged FluPol subunits. NLR signals were quantified as described in Figure 1B. Data are mean ± s.d. n = two technical replicates representative of two or three independent assays.

Finally, we investigated interactions between FluPol subunits (PA, PB1 or PB2) of the WSN strain (H1N1) and Pol II subunits including RPB2 to RPB12 (Figure 2A,C), and a fragment of the RPB1 CTD. As expected from structural analyses [14], the RPB1 CTD was found to interact with PA alone and with the assembled FluPol (Figure 2B). With other Pol II subunits, the range of NLR values was 1- to 2-logs of magnitude lower than those observed for internal Pol II and FluPol interactions (Figure 2C). For each tagged FluPol subunits, RPB3 and RPB4 (and to a less extent RPB7 and RPB8) yielded the highest NLR signals when in the presence of an intact FluPol complex. These data were indicative of proximity interactions between FluPol and Pol II not accounted for by RPB1 CTD (and might possibly involve RPB3 and RPB4). We noted further that, for the PA bait, the FluPol-Pol II interaction-signals benefited from co-expressed untagged PB1 but not from coexpression of untagged PB2. Likewise, NLR signals obtained with tagged PB1 and Pol II subunits remained unaffected upon co-expression of untagged PA and PB2. Finally, interactions of the PB2 bait with Pol II subunits were affected by the presence of other FluPol subunits only for RPB4 and RPB7. These data showed that interactions between FluPol and Pol II subunits were not strictly dependent on the assembly of FluPol into an intact complex, and suggested that the assay is amenable to analyze individually the implication of structurally-defined protein domains of FluPol.

### 3.2. The N-Terminal Region of PB2 Mediates Interaction with RPB4

**Figure 3:**
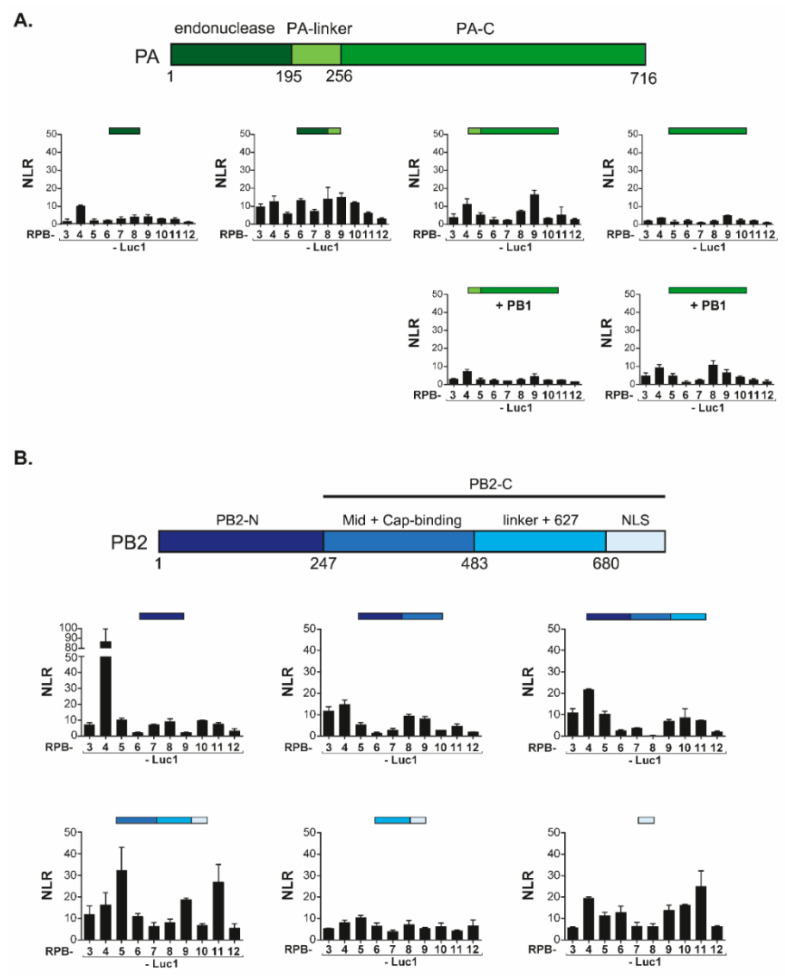
Split-luciferase assays between PA and PB2 domains of FluPol and Pol II subunits. (**A**) Luc1-PA domains and Luc2-subunits of Pol II were co-expressed into HEK293T cells along with PB1 or an empty plasmid. Twenty-four hours post-transfection, cells were lysed and luminescence was measured. NLR were determined as shown in Figure 1B. (**B**) Luc1-PB2 domains and Luc2-subunits of Pol II were co-expressed into HEK293T cells with NLR determined as shown in Figure 1B. Data are mean ± s.d. n = two technical replicates.

FluPol can be subdivided in functional and structural protein domains. It associates an invariant core comprising the PB1 subunit, stabilized by the PA-linker and the PA-C [amino acid 197 to the C-terminus] domain from the PA subunit, and the PB2-N [amino acid 1 to 247] from the PB2 subunit. This core is then associated with flexibly-linked peripheral domains including the PA endonuclease domain and the two-third C-terminal domain of PB2] [27]. To map the domains of FluPol principally involved in interactions with Pol II, we generated constructs driving expression of corresponding sub-fragments of PA and PB2. Most of these domains were shown to acquire their native fold when expressed without other FluPol components [2]. In complementation assays with the Pol II subunits, the PA-derived constructs co-expressed with PB1 did not reveal any clear association with Pol II subunits, all combinations yielding very low NLR signals (Figure 3A). In contrast, co-expression of the core-associated PA domains with a full set of Pol II subunits revealed a strong interaction (NLR score at 85) between PB2-N (residues 1 to 247) and RPB4 which was not observed with other Pol II subunits (Figure 3B). Furthermore, the PB2-C (residues 247 to 759) construct also yielded high NLR scores (approximately 30) with RPB5 and RPB11.

**Figure 4:**
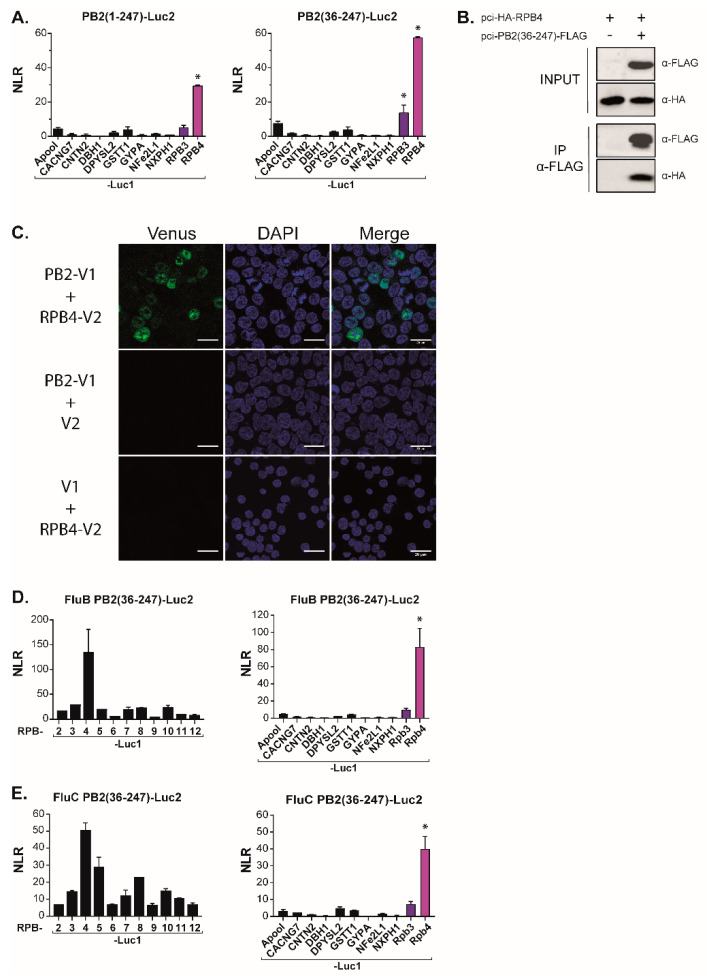
PB2-N domain interacts with the Pol II subunit RPB4. (**A**) Split-luciferase assays between PB2-N forms (Luc2-PB2(1−247) and Luc2-PB2(36−247) and Luc1-tagged irrelevant proteins(Apool for MICOS complex subunit MIC27, UniProtKB ID Q6UXV4; CACNG7 for Voltage-dependent calcium channel gamma-7 subunit, P62955; CNTN2 for Contactin-2, Q02246; DBH1 for dopamine beta-hydroxylase, P09172; DPYSL2 for dihydropyrimidinase-related protein 2, Q16555; GSTT1 for glutathione S-transferase theta-1, P30711; GYPA for glycophorin-A, P02724; NFE2L1 for endoplasmic reticulum membrane sensor NFE2L1, Q14494 and NXPH1 for Neurexophilin-1 for P58417). Data are mean ± s.d. n = two technical replicates. (**B**) Coimmunoprecipitation assay. Plasmids encoding HA-RPB4 and PB2(36−247)-FLAG were transfected in HEK 293T cells. Twenty-four hours post-transfection, cells were lysed, PB2(36−247)-FLAG was immunoprecipitated and RPB4 was revealed by Western Blotting. (**C**) Split-Venus complementation assay between PB2 and RPB4. HEK 293T cells were co-transfected with expression plasmids encoding PB2-Venus1 (PB2-V1), RPB4-Venus 2 (RPB4-V2) and/or with plasmids encoding Venus1 (V1) and Venus2 (V2) moieties. Cells were fixed 24 h later, nuclei were marked with DAPI and fluorescence emitted by reconstituted Venus was measured by confocal microscopy. Bars = 20 µm. (**D**,**E**) The interaction between PB2(36−247) and RPB4 is conserved in influenza types B and C. Plasmids encoding luc2-tagged forms of PB2(36−247) from influenza B (**D**) or influenza C (**E**) viruses were co-expressed with Luc1-tagged Pol II subunits in HEK 293T cells. Twenty-four hours post transfection, cells were lysed and luminescence was measured. NLR values were calculated as described in Figure 1. Data are means ± s.d. n = two technical replicates representative of two independent assays. * indicates outliers with a *p* < 0.01.

To validate the specificity of the PB2-N/RPB4 interaction, we next challenged the PB2-N construct with a set of proteins unrelated to Pol II. In these experiments, neither PB2-N, nor a shorter PB2(36–247) construct lacking 35 N-ter residues interacting with PB1 [28], displayed any interaction with the mock targets (Figure 4A). Together, these results were indicative of a specific contact between host Pol II subunit RPB4 and the PB2-N domain while also suggesting auxiliary contacts between RPB5 and RPB11 and the C-terminal regions of PB2.

### 3.3. The PB2RPB4 Interaction Domain Is Essential for the Virus Life Cycle

We next wished to probe the robustness and the biological relevance of the PB2/RPB4 interaction. Co-immunoprecipitation-western assays with epitope-tagged forms of RPB4 and of the PB2(36−247) construct allowed for further narrowing down the region of PB2 interacting with RPB4 while also demonstrating that the interaction resisted the relatively stringent conditions characterizing these assays (150 mM NaCl, 1% Triton X-100; Figure 4B). To further test the interaction under in vivo conditions, we carried out a second binary complementation assay, with PB2 and RPB4, respectively, tagged with the N- and C-moieties of the Venus fluorescent protein (Figure 4C). Co-transfection of these constructs into HEK293T cells resulted in a strong nuclear fluorescence signal, establishing that PB2 can interact with RPB4 under these conditions while also illustrating that this interaction occurs in the appropriate cellular compartment.

To investigate whether the PB2/RPB4 interaction was confirmed among different influenza virus types, we also constructed a luciferase complementation assay with the PB2 of influenza B and C viruses. In this assay, the influenza B PB2(36−247) construct interacted with RPB4 but not with any other Pol II subunit (nor with any of the unrelated target proteins described above (Figure 4D)). Likewise, the influenza C construct was associated with RPB4. However, it also yielded a signal with additional Pol II subunits, possibly due to indirect interactions (Figure 4E). In this assay, this influenza C PB2 domains did not display any interaction with the non-relevant control proteins.

**Figure 5:**
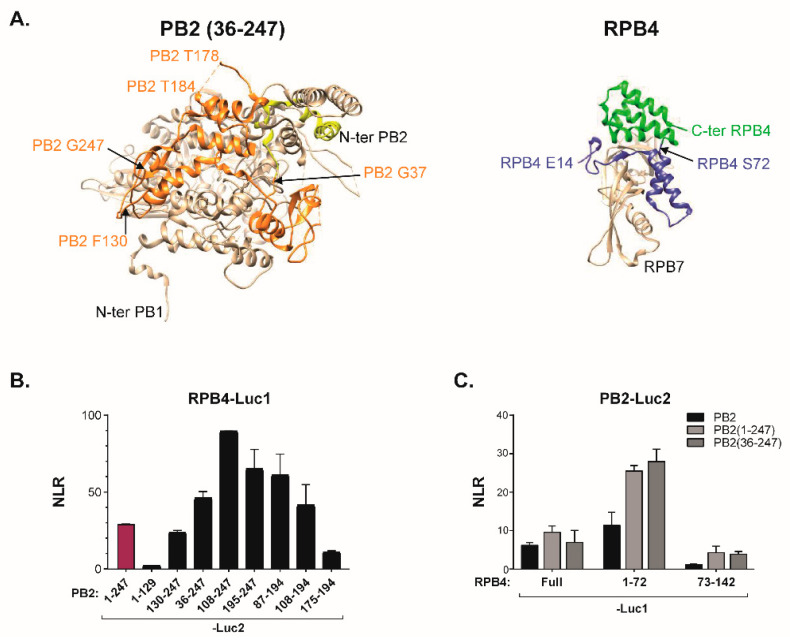
Identification of the PB2 and RPB4 amino acids stretches involved in the FluPol-RPB4 interaction. (**A**) Structures of the 36−247 domain of PB2 with the PB1 adjacent domain (PDB ID: 6QPF) and of the RPB4/RPB7 dimer (PDB ID: 2C35). (**B**,**C**) Split-luciferase complementation assay. (**B**) Luc2-tagged stretches of PB2(1−247) and Luc1-RPB4 were co-transfected into HEK293T cells. 24 h after transfection, cells were lysed and luminescence was measured. (**C**) The Luc1-tagged N- or C-moieties of RPB4 were co-expressed with the Luc2-tagged PB2 subunit or with N-ter derived forms of PB2 into HEK293T cells. After cell lysis, luminescence was measured and NLR calculated as in Figure 1B. Data are mean ± s.d. n = two technical replicates representative of three independents assays.

To further map regions involved in the PB2/RPB4 interaction, we next expressed a series of truncations of PB2-N. When tested in the split-luciferase system, several discrete regions of the PB2 amino acids, stretching from amino acid 130 to 247, yielded a signal with RPB4 (Figure 5). Reciprocally, truncations of RPB4 identified the N-ter moiety of this protein (amino acids 1−72) as essential for the interaction (Figure 5C). Interestingly, when mapped on the 3D-structures of FluPol and RPB4 [26,29], the protein regions identified in these two sets of deletion mutants matched a series of helixes located at the surface on the two proteins at a position highly compatible with an implication in protein–protein interactions.

**Figure 6:**
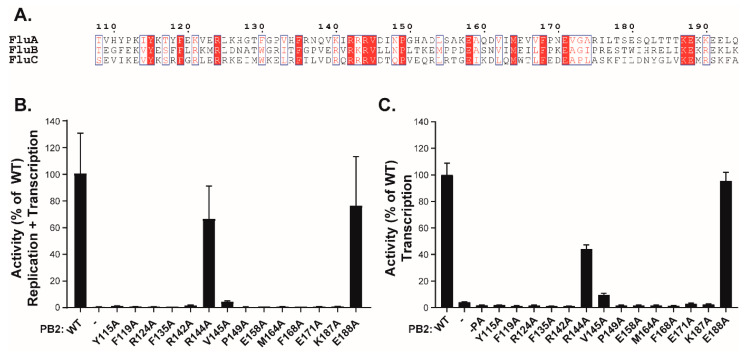
Transcription/replication activity of FluPol with PB2 mutants. (**A**) Multi-alignment with the PB2 domains (residues 108 to 194) of influenza A, B, and C viruses made with ESPript [30]. Strictly conserved residues are white on a red background, and partially conserved residues are red. (**B**) Plasmids expressing NP, PB1, PA, and wild type or PB2 mutants were cotransfected in 293T cells together with the reporter plasmid WSN-NA-firefly luciferase, allowing the quantification of the polymerase activity. In panel (**C**), the same procedure than in (**B**) was used, except that the PA C95A mutant deficient in replicase activity [15] was used instead of the wt PA subunit. Data are mean ± n = four technical replicates representative of two independent assays.

Finally, to address the biological function of the PB2 (130−247) region, we engineered a series of fourteen point-mutants on residues conserved among influenza A, B, and C viruses (Figure 6A). The activity of these mutants was then assessed in two assays, respectively measuring either the resultant of both replication and transcription (R+T) or just transcription (T) (Figure 6B,C). In the latter assay, wild-type PA was replaced by a C95A mutant, rendering FluPol defective for replication [22]. Both assays relied on a pPol1-WSN-NA-firefly luciferase plasmid producing a viral RNA (vRNA) in which the coding sequence of the NA gene was replaced by the firefly luciferase gene. This plasmid was transfected into HEK-293T cells together with expression vectors for the appropriate PA construct, the wild-type PB1 and NP constructs, and the various PB2 mutants. The luciferase enzymatic activity measured in extracts of transfected cells and reflecting either R+T or T activities dependent on the used PA construct (WT or C95A mutant), revealed that most of the PB2 mutations, with the exception of R144A and E188A, strongly decreased FluPol activity in both assays. These observations indicate that this region of PB2 and its conserved residues are critical for viral transcription (Figure 6C). Finally, to definitively establish the importance of the PB2-N region in the virus cycle, we selected residues 115, 142, and 144 for alanine substitution. Using plasmid-driven reverse genetics, we successfully recovered virus mutants for each of these substitutions. While the R144A virus was found to replicate at high titers (1.2 × 10^7^ pfu/mL in cell culture medium after transfection), the Y115A and the R142A mutants replicated poorly (6 × 10^4^ pfu/mL), in accordance with the results obtained in the replication/transcription assays.

## 4. Discussion

The interaction between FluPol and the Pol II complex through the CTD of its RPB1 subunit has been well-established both functionally and structurally [9]. The interplay between the two polymerases has been recognized to allow cap-snatching on nascent host mRNAs then priming of the viral mRNA synthesis. However, the question concerning whether the docking of FluPol to the nascent capped RNA relied solely on RBP1 or involved additional protein-protein interactions had never been elucidated. In this study, we used in vitro binary complementation assays to screen for auxiliary interactions between FluPol and Pol II subunits. We found that, in addition to RPB1, FluPol associated with RPB4, one of the twelve Pol II subunits, which is structurally connected to the core of Pol II via RPB7. This interaction, which was confirmed in co-immunoprecipitation assays, was mapped to the N-terminal third of PB2 and was found to be conserved among influenza B and C subtypes. Together, these observations suggest a critical role for multiple FluPol-Pol II interactions in the life cycle of IAVs.

The RPB4/RPB7 heterodimer forms a stalk-like protrusion from the Pol II core complex below a mobile clamp domain, adjacent to the CTD of RPB1 and the RNA exit channel, a position with potential for interactions with multiple components such as CTD-bound factors and modifying enzymes and emerging nascent RNAs [31,32,33,34,35,36,37]. In fact, the RPB4/RPB7 stalk has been proposed to act as an additional landing platform for transcription-related factors, some of which also interact with the CTD [38,39]. It is therefore tempting to speculate that the binding of FluPol to RPB4 synergizes with the CTD-mediated interaction to target nascent capped RNAs.

**Figure 7:**
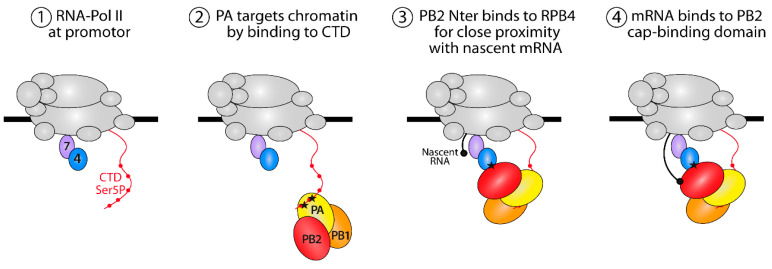
Proposed model for the interaction interplay between FluPol and Pol II. Pol II is represented in gray with RPB4 and RPB7 subunits in blue and lavender, respectively. FluPol first recognizes the YSPTSpPS motifs (with the serine phosphorylated at position 5 indicated by red dot) in the CTD of RPB1 to bind the RPB4 subunit located at the vicinity of the Pol II pore which extrudes nascent mRNA during transcription. PB2 binds the cap (black dot) of the mRNA by its cap-binding domain. Black asterisks indicate FluPol binding sites on the Pol II CTD and RPB4.

Despite low sequence similarity between the N-terminal third of PB2 among influenza A, B and C viruses, these homologous domains were all found to interact with the RPB4 subunit. When mutating conserved residues in this domain, twelve of fourteen substitutions deeply impacted the transcription activity of FluPol. This documented the functional implication of the N-ter PB2 domain in reaching an efficient interaction with the host transcription machinery. Therefore, we propose a model (Figure 7) in which FluPol first targets Pol II via its PA-CTD interaction and then binds the RPB4 subunit via PB2 to edge closer to the RNA exit tunnel of Pol II. This would bring the cap-binding domain of PB2 in close proximity to nascent mRNAs, thereby allowing the cap-snatching by FluPol to occur.

What could the time-line of the FluPol-RPB4/RPB7 dimer interaction be? Nascent transcript capping occurs immediately after the emergence of the 5′-end triphosphate from the RNA exit tunnel and is concomitant with promoter-proximal pausing of RNA Pol II bound to NELF and DSIF [40,41]. Shortly after cap completion, the interaction of the cap-binding complex (CBC) with a NELF subunit correlates with its targeting to the paused RNA Pol II complex and increases by 100–200-fold its affinity for the modified 5′ end of the transcript. Since the CBC has an affinity for the cap structure that is higher than that of FluPol, we propose that FluPol binding to Pol II via RPB1 CTD and RPB4/RPB7 interferes with CBC recruitment or with its access to nascent 5′-capped Pol II transcripts. Alternatively, FluPol may inhibit the binding of NELF to Pol II or its association with CBC. These hypotheses are currently under investigation and will allow for better understanding of how FluPol efficiently controls capping of viral mRNAs.

## Data Availability

Data supporting reported results can be provided by B.D.
